# Clinical and Radiological Outcomes of Angiographically Proven Central Nervous System Arteriopathy

**DOI:** 10.7759/cureus.77897

**Published:** 2025-01-23

**Authors:** Javeria Raza Alvi, Saher Gul Ahdi, Narjis Fatima Alvi, Muhammad Zia Ur Rehman, Amna Tariq, Tipu Sultan

**Affiliations:** 1 Pediatric Neurology, University of Child Health Sciences, The Children's Hospital, Lahore, PAK; 2 Physical Medicine and Rehabilitation, University of Child Health Sciences, The Children's Hospital, Lahore, PAK; 3 Pediatric Radiology, University of Child Health Sciences, The Children's Hospital, Lahore, PAK

**Keywords:** arterial ischemic stroke, childhood stroke, magnetic resonance angiography, motor outcome, moyamoya disease

## Abstract

Objective: Childhood stroke is a significant contributor to neurological morbidity often leading to debilitating outcomes. This study aimed to assess the clinical and radiological outcomes of angiographically proven central nervous system (CNS) arteriopathy in children.

Material and methods: This prospective cohort study was conducted from June 2023 to May 2024 at The Children’s Hospital, Lahore, Pakistan. The clinical outcomes were assessed using the Pediatric National Institute of Health Stroke Scale (PedNIHSS) at baseline and compared at six-month follow-up, while radiological outcomes were categorized as stable, regressive, or progressive disease. Clinical presentation, etiology, baseline PedNIHSS score, and arterial involvement were correlated with motor outcomes.

Results: Out of the 38 patients, 63.2% were female patients with a mean age of 4.27±2.43 years. The most common age group was 2-5 years (50.0%), followed by >5-10 years (31.6%). Global clinical presentation was seen in 60.5%, while 29.0% had a recurrence of stroke. Moyamoya disease (21.1%) was the most common etiological factor, followed by primary CNS angiitis (13.0%), infections (8.0%), and post-varicella focal cerebral arteriopathy (8.0%). Bilateral infarcts (55.3%) and anterior circulation involvement (68.4%) were the most affected areas, with the middle cerebral artery (MCA) being the most affected with complete involvement in 18.4% and partial involvement in 81.5% of cases. The mean baseline PedNIHSS score was 29±6.4, which improved to 19±4.8 at the six-month follow-up. Radiological outcomes showed in 42.2% of cases, with a progression of the disease on follow-up MRA; 31.6% had stable disease, while 21.0% had regressive disease. Significant factors associated with poor motor outcomes included global presentation (p=0.000008), etiological factors (p=0.047), bilateral infarcts (p=0.050), severe baseline PedNIHSS (p=0.000019), and progressive radiological disease (p=0.003).

Conclusion: This study highlights the significant neurological impact of pediatric stroke. Early diagnosis, investigation of underlying causes, and identification of recurrence risk factors are crucial in preventing immediate and long-term complications.

## Introduction

Childhood arterial ischemic stroke (AIS) affects 1.0 to 7.9 per 100,000 children beyond the neonatal period and is a major contributor to long-term disability [1]. Most prevalent are motor deficits with hemiparesis, reported in up to 75% of patients [2]. Arteriopathy is a term used to describe any disease affecting the arteries. It is an umbrella term that covers vascular abnormalities caused by degenerative, metabolic, inflammatory conditions, embolic diseases, coagulation disorders, and non-inflammatory progressive disorders [1].

AIS in children differs significantly from adults in terms of etiology and clinical presentation. Disease course can be acute to insidious with clinical features highly variable in children, including headache, altered cognition, focal neurologic deficits (e.g., hemiparesis, ataxia, aphasia, dysarthria, and visual disturbances), seizures, and encephalopathy [3]. On the basis of radiological findings, central nervous system (CNS) arteriopathies are divided into two categories, namely angiographic positive and angiographic negative arteriopathies [4]. Our main subject of concern is angiographic positive arteriopathies, which comprise a heterogeneous group of disorders, including various non-inflammatory and inflammatory etiologies [5]. Radiological features of CNS arteriopathy on angiography may include unilateral or bilateral steno-occlusive vascular lesions involving proximal or distal branches of the internal carotid artery, major branches such as the middle cerebral artery (MCA) and anterior cerebral artery (ACA), or the posterior vertebrobasilar arteries [4]. Furthermore, the conversion of focal cerebral arteriopathy to the progressive type and multivessel arteriopathy to moyamoya could only be described with follow-up neuroimaging.

The rationale of this study was to review local data regarding the clinical and radiological outcomes of CNS arteriopathy. It also contributed to reporting the local incidence, etiology, and clinical presentation of different arteriopathies, providing insights that could lead to an organized approach toward the identification of these arteriopathies, which had been underreported in our setup previously. Therefore, we conducted this study to evaluate the clinical and radiological outcomes of angiographically proven arteriopathy in patients who presented to the Pediatric Neurology department of a tertiary care teaching hospital.

## Materials and methods

This prospective cohort study was conducted from June 2023 to May 2024 at the inpatient and outpatient department of Pediatric Neurology, University of Child Health Sciences, The Children’s Hospital, Lahore, Pakistan, after obtaining ethical approval from the Institutional Review Board (approval number 2021-414-CHICH dated 29-07-2021). The sample size was calculated to be 38 cases using OpenEpi (www.OpenEpi.com), taking an estimated incidence of 2.5% of pediatric AIS with a 95% confidence interval and 5% margin of error [1].

All children having an acute neurological deficit with AIS and angiographically proven vasculopathy, aged >1 month to 15 years, were included in the study. Patients with small vessel vasculitis with negative angiography or patients having stroke without any territorial involvement on magnetic resonance imaging (MRI) were excluded. Angiographically proven arteriopathy was defined as abnormalities detected on magnetic resonance angiography (MRA), including vessel narrowing, beading, or irregularities in the vessel wall [6]. The outcome was defined in terms of motor and radiological parameters. For motor outcome, the Pediatric National Institutes of Health Stroke Scale (PedNIHSS) was used as it is a highly effective tool for evaluating pediatric stroke across multiple domains, and it can be trended over time to assess recovery. The score was further classified into five groups: 0 = no stroke, 1-4 = mild stroke, 5-15 = moderate stroke, 15-20 = moderately severe stroke, and 21-42 = severe stroke. Additionally, it was categorized as a good (no or mild stroke) or poor (moderate to severe stroke) outcome [7]. The radiological outcome was measured as a stable, regressive, or progressive disease [8]. Arterial involvement was categorized as partial or complete, depending on the extent of involvement. Partial was defined when there was involvement of either superior or inferior division, whereas complete was defined as occlusion of stem leading to infarction of its entire territory. 

To determine etiology, cerebrospinal fluid analysis (when indicated), hematological, cardiac, and metabolic testing, and other relevant investigations (antibody titers) were conducted. The etiologies were categorized as follows: infectious (post-varicella arteriopathy, meningitis), hematologic/thrombotic (hemoglobinopathy, antiphospholipid antibody syndrome, inherited protein S, protein C, antithrombin III deficiency, factor V Leiden mutation, hyperhomocysteinemia, and anemia), inflammatory (primary CNS vasculitis), systemic inflammatory or autoimmune disease, cardioembolic, genetic vasculopathy (trisomy 21, neurofibromatosis), metabolic (mitochondrial disorders including mitochondrial encephalomyopathy, lactic acidosis, and stroke-like episodes (MELAS) and complex 1 deficiency), and non-inflammatory intracranial vasculopathy (moyamoya disease).

An informed consent was obtained from the primary caregiver of the participating child. Data were collected using a pre-designed proforma, recording details such as age at onset, gender, clinical presentation, risk factors and etiology, PedNIHSS score, and MRA findings at baseline and six-month follow-up. Since multiple risk factors may contribute to a poor outcome, they were coded as single/multiple. Clinical presentation was coded as global (reduced level of consciousness, generalized seizures, headache) and focal (hemiparesis, visual field/speech deficit, focal seizures) [2]. Data analysis was performed using IBM SPSS Statistics version 25 (IBM Corp., Armonk, US). Variables such as age at onset and PedNIHSS score, at baseline and six months, were expressed as mean and standard deviation. Categorical variables were expressed as frequency in percentage. The chi-square and Fisher's exact test were employed to assess associations between outcomes and variables, including age, etiology, risk factors, recurrence, and arterial involvement. Regression analysis and analysis of variance (ANOVA) were applied to determine the effects of etiology, risk factors, recurrence, and arterial involvement on motor outcome. A p-value of ≤0.05 was considered statistically significant.

## Results

**Figure 1:**
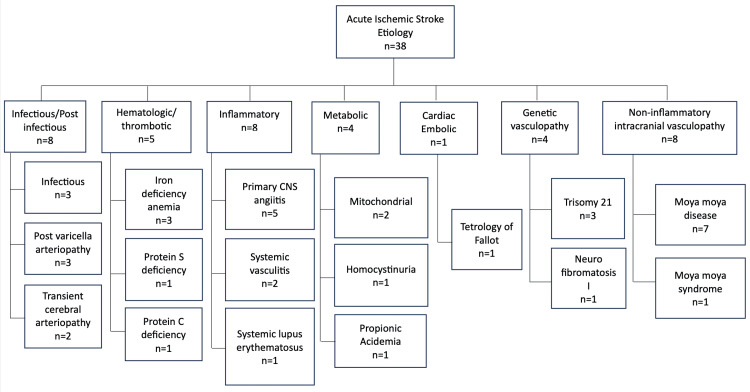
A breakdown of etiological factors CNS: Central nervous system The figure was derived from the statistical analyses conducted by the authors.

The study included 38 patients with AIS, out of which 24 (63.2%) were female patients with a mean age of 4.27±2.43 years. Table 1 shows their background and clinical characteristics, including distribution across different age groups, common clinical presentation, risk factors, and radiological findings. Hemiparesis was the most predominant feature in 38 (100%) patients, followed by central facial palsy in 17 (44.7%), dysarthria in 14 (36.8%), and visual field disturbances in four (10.5%); however, reduced level of consciousness was seen in 21 (55.2%) patients. Recurrent stroke was seen in 11 (28.9%) patients. A breakdown of etiological factors is presented in Figure 1.

**Table 1:** Distribution of study participants by background, clinical characteristics, and radiological findings * In many cases, the territories of MCA, ACA, and PCA were overlapping, so each artery’s individual involvement is documented here. MCA: Middle cerebral artery; ACA: Anterior cerebral artery; PCA: Posterior cerebral artery

Characteristics	Frequency, n (%)
Gender	
Male	14 (36.8%)
Female	24 (63.2%)
Age in groups	
>1 month to 1 year	3 (7.9%)
>1-2 years	3 (7.9%)
>2-5 years	19 (50.0%)
>5-10 years	12 (31.6%)
>10-15 years	1 (2.6%)
Clinical presentation	
Global	23 (60.5%)
Focal	15 (39.5%)
Recurrence of stroke	
Yes	11 (29.0%)
No	27 (71.0%)
Risk factors	
Single	22 (57.9%)
Multiple	16 (42.1%)
Neuroimaging findings	
Distribution of lesion	
Anterior	26 (68.4%)
Posterior	1 (2.6%)
Both	11 (29.0%)
Site of infarct	
Left	11 (29.0%)
Right	9 (23.6%)
Both	18 (47.4%)
Vascular territory*	
MCA (Complete)	7 (18.4%)
MCA (Partial)	31 (81.5%)
ACA	26 (68.4%)
PCA	4 (10.5%)
Collaterals	
Yes	11 (29.0%)
No	27 (71.0%)
Radiological outcome	
Stable	12 (31.6%)
Regressive	8 (21.0%)
Progressive	16 (42.2%)
Different site involvement	1 (2.6%)
Indeterminate	1 (2.6%)

The neuroimaging findings showed that the left side (29.0%) was more commonly involved than the right side (23.6%), predominantly in the anterior circulation (68.4%), with MCA emerging as the most frequently affected vascular territory in isolation in 14 patients (36.8%) and simultaneously with other arteries (ACA and posterior cerebral artery (PCA)) in 24 patients (63.2%). Furthermore, there was complete involvement of MCA in 18.4% of cases and partial involvement in 81.5% of cases. When MCA was partially involved in isolation, five patients (13.2%) had focal symptoms and two (5.3%) had global symptoms. But, when it was completely involved or simultaneously involved with other arteries, the global presentation was 21 (55.3%), although it was not statistically significant (p=0.156). The radiological outcome is described in Table 1. Out of 16 (42.2%) patients having a progressive disease, eight (21.1%) had progressed to moyamoya disease. One patient did not have follow-up imaging, so the outcome was indeterminate.

**Figure 2:**
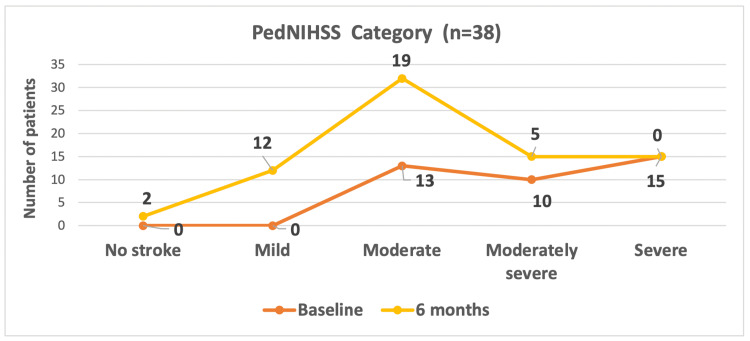
PedNIHSS category at baseline and six-month follow up PedNIHSS: Pediatric National Institutes of Health Stroke Scale The figure was derived from the statistical analyses conducted by the authors.

The mean PedNIHSS score at baseline was 29±6.4, which decreased to 19±4.8 at the six-month follow-up. PedNIHSS category at baseline and six-month follow-up is shown in Figure 2. It depicts that at baseline, 13 (34.2%) patients had a moderate stroke, 10 (26.3%) had a moderately severe stroke, and 15 (39.5%) had a severe stroke. By the six-month follow-up, the neurological status improved, with two (5.3%) having no stroke, 12 (31.5%) classified as mild, 19 (50.0%) as moderate, and five (13.2%) as moderately severe (Figure 2). With regards to the outcome, good outcome (no to mild category) was seen in 14 (36.8%) patients, while poor outcome (moderate to severe category) was seen in 24 (63.2%) patients.

Table 2 outlines the factors influencing the motor outcome, including clinical presentation, risk factors, recurrent attacks, PedNIHSS category at baseline, arterial involvement, and stroke location. We found global clinical presentation (0.000008), etiology (0.047), bilateral distribution of infarcts (0.050), severe baseline PedNIHSS score (0.000019), and progressive disease on radiology (0.003) to be statistically significant and linked with poor motor outcome.

**Table 2:** Factors influencing motor outcomes ^a ^p-value calculated using chi-square. ^b ^p-value calculated using Fisher’s exact test. A p-value of ≤0.05 was considered statistically significant. PedNIHSS: Pediatric National Institutes of Health Stroke Scale

Characteristics	Good outcome	Poor outcome	p-value
	(n=14)	(n=24)	
Clinical presentation			
Global	2 (14.3%)	21 (87.5%)	0.000008^a^
Focal	12 (85.7%)	3 (12.5%)	
Recurrence of stroke			
Yes	2 (14.3%)	9 (37.5%)	0.128^a^
No	12 (85.7%)	15 (62.5%)	
Etiology in groups			
Infectious	6 (42.9%)	2 (8.3%)	
Hematologic/thrombotic	1 (7.1%)	4 (16.6%)	
Inflammatory	2 (14.3%)	6 (25.0%)	
Metabolic	0 (0.0%)	4 (16.7%)	0.047^a^
Cardioembolic	0 (0.0%)	1 (4.2%)	
Genetic vasculopathy	3 (21.4%)	1 (4.2%)	
Moyamoya disease	2 (14.3%)	6 (25.0%)	
Risk factors			
Single	10 (71.4%)	12 (50.0%)	
Multiple	4 (28.5%)	12 (50.0%)	0.197^a^
Arterial circulation			
Anterior	10 (71.4%)	16 (66.6%)	
Posterior	0 (0.0%)	1 (4.2%)	0.736^a^
Both	4 (28.6%)	7 (29.2%)	
Site of infarct			
Left	6 (42.9%)	5 (20.8%)	
Right	5 (35.7%)	4 (16.7%)	0.050^a^
Both	3 (21.4%)	15 (62.5%)	
Baseline PedNIHSS category			
Moderate	11 (78.6%)	2 (8.3%)	
Moderate to severe	3 (21.4%)	7 (29.2%)	0.000019^a^
Severe	0 (0.0%)	15 (62.5%)	
Radiological outcome	(n=13)	(n=24)	
Stable	6 (46.2%)	6 (25.0%)	
Regressive	4 (30.8%)	4 (16.7%)	0.037^a^
Progressive	2 (15.4%)	14 (58.3%)	0.003^b^
Different site involvement	1 (7.6%)	0 (0.0%)	

**Figure 3:**
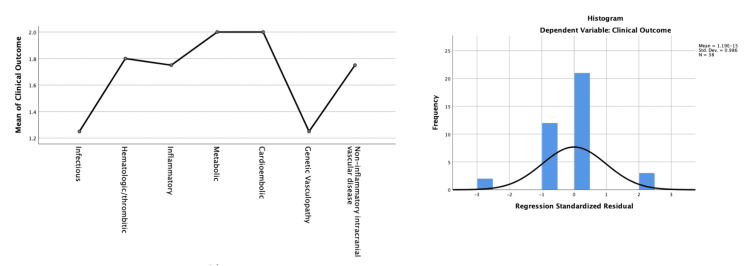
Mean clinical motor outcomes across various etiologies (left) and a histogram of regression standardized residuals for clinical motor outcomes (right) The figure was derived from the statistical analyses conducted by the authors.

A logistic regression analysis was applied to evaluate the factors influencing poor motor outcomes compared to good outcomes (Table 3). Figure 3 shows the mean clinical outcomes across various etiologies and a histogram of regression standardized residuals for clinical motor outcomes. Clinical presentation with global type was associated with poor outcome scores, suggesting it to be a strong predictor (0.001). Moreover, ANOVA was applied to determine the differences in clinical outcomes (good vs. poor) based on the predictor variables, which revealed a statistically significant difference between groups F (1,36)=13.322, p=0.001.

**Table 3:** Logistic regression analysis of factors influencing poor motor stroke outcomes ^a ^p-value calculated using logistic regression. A p-value of ≤0.05 was considered statistically significant.

Variables	Odds ratio (CI)	p-value^ a^
Gender	0.745 (95% CI: 0.053–10.531)	0.828
Age at onset of stroke	1.001 (95% CI: 0.630–1.589)	0.998
Clinical presentation	0.009 (95% CI: 0.001–0.155)	0.001
Recurrence of stroke	0.109 (95% CI: 0.004–3.030)	0.191
Etiology	1.169 (95% CI: 0.741–1.843)	0.502

## Discussion

Childhood stroke is a significant cause of neurological impairment, resulting in severe and lasting outcomes. In our cohort, we found considerable improvement in the PedNIHSS score from 29±6.4 to 19±4.8 at the six-month follow-up. Despite a significant shift in stroke outcomes from severe to mild and moderate categories, there still persisted residual deficits that continued to have a significant motor impact. This aligns with the study done by Hidalgo et al. and Beslow et al., wherein PedNIHSS scores were between mild to moderate categories [6,9]. The persistence of residual deficits despite improvement from baseline underscores the need for vigorous and sustained rehabilitation to optimize motor function and prevent secondary complications.

Regarding the radiological outcome, 43.2% of patients had progression with 21.1% developing moyamoya disease, while 32.4% had stable disease. In the cohort studied by Lee et al., moyamoya accounted for about 8% of childhood AIS and was associated with an increased risk of recurrent stroke [10]. The recurrence of stroke in our cohort was seen in 29.0% of cases, notably in patients with moyamoya disease and syndrome (neurofibromatosis 1 (NF1)) but also in inflammatory conditions, including systemic lupus erythematosus and primary CNS angiitis, similar to the study done by Akter et al. [11]. Moreover, the progressive changes in follow-up MRA were strongly associated with poorer clinical outcomes (p=0.03). Radiological regression occurred in 21.6% of cases while stable disease was seen in 32.4% of cases. Out of these, post-varicella focal cerebral arteriopathy (8.0%) and transient cerebral arteriopathy (5.0%) were the most common diseases to improve in our cohort. The reported prevalence of FCA varies across studies, with Akter et al. identifying only 9% of patients with FCA, aligning with our cases, whereas Goktas et al. cohort had reported 46% of cases, higher than ours [11,12]. These discrepancies could be due to several factors including environmental and genetic variability, timing of diagnosis, or differences in the study population.

In our study, we found a higher prevalence of stroke in females compared to males, which contrasts with the findings of most studies that report a male predominance [13-15]. However, the study done by Ghani et al. in the same region also demonstrated a higher prevalence in females [16]. This divergence emphasizes the need for further studies into potential gender-related risk factors, including hormonal, genetic, and environmental factors. The mean age of patients in our cohort was 4.27±2.43 with 50% of patients between the 2-5 years age group. These findings align with the results of Ghani et al. and Amlie-Lefond et al. [16,17]. However, they contrast with the findings of Rafay et al. who identified the 6-9 years age group as being the most affected by arteriopathy [17]. Global clinical presentation (60.5%) was more common than the focal one (39.5%) as compared to other cohorts [2,6], but the most prevalent clinical feature found in both global and focal presentation was hemiplegia consistent with other cohorts [12,16,18]. When comparing the clinical presentation with stroke outcome (good vs. poor), we found that global presentation was strongly associated with poorer outcomes (0.000008), with residual deficits at follow-up. This may be primarily attributed to a larger or multiple vessel involvement and diffuse ischemic injury, which carry a worse prognosis. Thus, early recognition of global symptoms as being the cause of stroke can guide aggressive treatment strategies for better outcomes.

Regarding the etiology, we found moyamoya disease (21.1%) as the most prevalent cause of arteriopathy in our cohort, followed by primary CNS angiitis (13.0%), infections (8.0%), and post-varicella focal cerebral arteriopathy (8.0%). While analyzing the etiology by gender, we found moyamoya disease more prevalent in males, while inflammatory etiology was more common in females. Studies done by Rafay et al. and Böhmer et al. also suggested arteriopathy as the most common cause of AIS, with moyamoya and focal cerebral arteriopathy being the most prominent subtypes [18-20]. However, our cohort differs in the second most common cause being primary CNS angiitis rather than post-varicella arteriopathy. This could be attributed to regional differences or environmental triggers with a higher burden of inflammatory response in our population.

When MRA findings were assessed for their location, arterial involvement, and laterality, we found bilateral involvement in 47.4% and left-sided in 29.0%. Anterior circulation was most commonly affected in 68.5% of cases, while 29% had both anterior and posterior circulation involvement with MCA being the most commonly involved artery. This aligns with the 2003-2014 International Pediatric Stroke Study (IPSS) data that reported anterior circulation to be involved in 67% of cases, and 25% had both anterior and posterior involvement [19]. Though Goktas et al. cohort had no cases with combined anterior and posterior involvement, the left side was predominantly involved similar to our cohort [12]. MCA remained the most commonly affected territory across multiple cohorts including ours [7,21-23].

Limitations

A smaller sample size and a single-center design might limit the applicability of results to other settings with differences in demographics and healthcare facilities.

## Conclusions

Stroke in children is a relatively common occurrence in our country; however, limited awareness regarding its presentation and potential causes often leads to delayed diagnosis, which complicates the overall prognosis. Our study underscores the considerable neurological impact of pediatric stroke. Despite significant improvement in PedNIHSS scores at the six-month follow-up, residual motor deficits persisted. We observed a higher prevalence of stroke in females compared to males. Moyamoya disease and primary CNS angiitis were identified as the most common causes, with radiological progression observed in moyamoya cases, which were linked to recurrent strokes. Bilateral involvement and anterior circulation were the most frequent patterns of arterial involvement in MRA. It is essential to thoroughly diagnose and investigate the underlying causes and identify risk factors for recurrence in order to prevent both immediate and long-term complications.
